# *Enterobacteriaceae* in Sewage Sludge and Digestate Intended for Soil Fertilization

**DOI:** 10.3390/pathogens13121056

**Published:** 2024-11-30

**Authors:** Angelina Wójcik-Fatla, Ewelina Farian, Katarzyna Kowalczyk, Jacek Sroka, Piotr Skowron, Grzegorz Siebielec, Jolanta Małgorzata Zdybel, Tamara Jadczyszyn, Tomasz Cencek

**Affiliations:** 1 Department of Health Biohazards and Parasitology, Institute of Rural Health, Jaczewskiego 2, 20-090 Lublin, Poland; farian.ewelina@imw.lublin.pl (E.F.); kowalczyk.katarzyna@imw.lublin.pl (K.K.); 2 Department of Parasitology and Invasive Diseases, National Veterinary Research Institute-State Research Institute, Aleja Partyzantów 57, 24-100 Puławy, Poland; jacek.sroka@piwet.pulawy.pl (J.S.); j.zdybel@piwet.pulawy.pl (J.M.Z.); tcencek@piwet.pulawy.pl (T.C.); 3 Department of Plant Nutrition and Fertilization, Institute of Soil Science and Plant Cultivation State Research Institute, Czartoryskich 8, 24-100 Puławy, Poland; pskowron@iung.pulawy.pl (P.S.); tj@iung.pulawy.pl (T.J.); 4 Department of Soil Science Erosion and Land Protection, Institute of Soil Science and Plant Cultivation-State Research Institute, Czartoryskich 8, 24-100 Puławy, Poland; gs@iung.pulawy.pl

**Keywords:** *Enterobacteriaceae*, soil, sewage sludge, digestate, *Escherichia coli*, *Salmonella*, fertilizers

## Abstract

Substances of organic origin are seeing increasing use in agriculture as rich sources of nutrients for plants. The aim of this study was to determine the microbiological contamination of sewage sludge and digestate to assess their safe use as fertilizers in Poland. The assessment of microbial soil, sewage sludge and digestate contamination was based on the total number of mesophilic bacteria and Gram-negative bacteria from the *Enterobacteriaceae* family. The presence of *Escherichia coli* and *Salmonella* spp. was identified via culture and the presence of *Enterobacteriaceae* species was determined via biochemical and molecular methods. In laboratory conditions, the survival of *E. coli* in soil fertilized with sewage sludge or digestate inoculated with a reference strain was determined. The average concentration of *Enterobacteriaceae* in soil, sewage sludge and digestate samples was 1.1 × 10^4^ CFU/g, 9.4 × 10^5^ CFU/g and 5.6 × 10^6^ CFU/g, respectively. *Escherichia coli* was detected in all sample types. From the soil samples, *Serratia*, *Enterobacter*, *Pantoea*, *Citrobacter* and *Pseudomonas* genera were identified the most frequently, while in sewage sludge and digestate, *E. coli* was predominant. Based on the results of our laboratory experiment, it can be concluded that after three weeks, fertilization with organic waste in acceptable doses does not significantly increase soil contamination with *Enterobacteriaceae*.

## 1. Introduction

Soil is a basic reservoir of microorganisms (e.g., bacteria, fungi, viruses, parasites), the diversity of which is the source of mechanisms regulating the impact of pathogens on other organisms [1]. The presence of indicator microorganisms in soil not only reflects the degree of contamination of the soil environment, but also provides information about the potential risk of contamination of agricultural produce and potential threats to human and animal health. In addition to naturally occurring microflora, the soil environment may also contain microorganisms introduced by improper sewage management or the use of contaminated manure, slurry or sewage sludge in agriculture.

Organic wastes contain plant nutrient contents and can be used to fertilize and improve soil properties in both raw and processed forms. Organic wastes are also introduced into the market as fertilizers and soil improvers, provided they meet quality requirements and contamination does not exceed permissible levels. In some cases, agricultural tests are necessary to confirm the suitability of the fertilizer for application to plants or for soil remediation. In addition to several quality parameters regarding minimum nutrient content and maximum heavy metal content, organic wastes and fertilizers cannot exceed the permissible values of biological contamination. Often, despite meeting all other quality requirements, organic wastes and fertilizers are disqualified from use because of the content of bacteria and parasite eggs [2].

In Poland, in accordance with the Regulation of the Minister of the Environment (2015) [3], sewage sludge may be used in agriculture and for land reclamation for agricultural purposes if *Salmonella* bacteria have not been isolated in a representative 100 g sludge sample and the total number of live eggs of intestinal parasites (e.g., *Toxocara* sp., *Trichuris* sp. *Ascaris* sp.) in 1 kg of dry matter is 0. There are no regulations requiring testing the concentration of *Enterobacteriaceae* bacteria or *Escherichia coli* as a representative species. In Europe, the Council Directive 86/278/EEC of June 1986 on the application of agricultural sewage sludge in agriculture [4] is still in force; while this directive establishes limit values for heavy metal concentrations, it does not provide indicators of biological origin. The presence of *E. coli* is a mandatory indicator of biological contamination of sewage sludge in only a few countries, such as Finland, Portugal and Lithuania, in amounts not exceeding 1000 CFU/g (colony-forming units per gram) or in no more than 100 CFU/g in Austria [5].

According to research by Estrada et al. [6], 80 days after the introduction of sewage sludge into the soil, the concentrations of most *Enterobacteriaceae*, *E. coli* and fecal coliform bacteria were below the detection limits in various conditions. Research conducted in Poland by Stańczyk-Mazanek and Stępniak [7] confirmed that the use of sewage sludge in doses not exceeding 40 t/ha should not cause soil contamination, but the use of higher doses may pose such a risk, especially from drug-resistant bacterial species. In turn, Michelon et al. [8] pointed out the need to limit and control enteric pathogens in organic substances introduced into the soil. The use of natural fertilizers should also consider the regional context, so that the introduced sewage does not present too much of a burden on the environment and result in, e.g., contamination of water bodies.

In terms of testing the number of *Enterobacteriaceae* (with a limit below 1000 CFU/g), Poland was subject, until 2024, to the provisions of the Regulation of the Minister of Agriculture and Rural Development (2008) [9] in the field of organic and organic–mineral fertilizers based on animal by-products. Pursuant to the 2007 Act on fertilizers and fertilization [10], digestate belongs to the group of manufactured fertilizers or fertilizers containing animal-derived products or by-products. Pursuant to the new ministerial regulation of August 2024 [11], fertilizers, plant cultivation aid products and post-fermentation products cannot contain live eggs of intestinal parasites and *Salmonella*, while the *Enterobacteriaceae* indicator has been removed.

In the current work, we focused exclusively on the contamination of soil, sewage sludge and digestate with bacteria from the *Enterobacteriaceae* family, and in particular, its representatives *E. coli* and *Salmonella*. The aim of the study was to determine the biodiversity of bacteria from the *Enterobacteriaceae* family in the tested samples and to determine safe limits of microbiological contamination of sludge and digestate based on an analysis of the risk of transfer of these pathogens to the soil in laboratory conditions. The results of this stage of the project will be used to verify current standards concerning regulations regarding the content of pathogenic bacteria in substances of organic origin intended for use as fertilizers in a way that does not pose a threat to human and animal health.

## 2. Materials and Methods

### 2.1. Determination of Bacteria Concentration Using Culture-Based Methods

#### 2.1.1. Sample Collection

Samples of arable soil (82), sewage sludge (9) and digestate (9) were collected for microbiological examination in 2021. Soil samples from agricultural fields in northeastern Poland were gathered from the top layer (up to 20 cm depth) by a soil stick sampler. In accordance with the principles of soil sampling, at least 10 punctures were made to obtain an average sample. The sewage sludge and digestate samples were obtained from biological wastewater treatment plants and agriculture biogas plants, respectively. The samples were intended for testing immediately after their delivery to the laboratory. The soil samples were sieved through a sieve with a hole diameter of 2 mm.

#### 2.1.2. Microbiological Culture

The assessment of microbial soil, sludge and digestate contamination was based on the following tests: total number of mesophilic bacteria, total number of Gram-negative bacteria from the *Enterobacteriaceae* family and presence of *E. coli* and *Salmonella* spp. Due to the lack of applicable procedures concerning microbiological testing of sewage sludge and digestates, the Polish standards pertaining to soil, food and feed research were used. Detection of *Salmonella* was performed according to the standard PN-Z-19000-1/2001 [12], *Escherichia coli* according to PN-EN ISO 16649-2:2004 [13], the total number of bacteria according to PN-EN ISO 4833-2:2013-12/AC [14] and *Enterobacteriaceae* according to PN-EN ISO 21528-2:2017-08 [15].

Two 10 g subsamples were taken from each sample for testing. One of the subsamples was suspended in 90 mL of Ringer’s solution, homogenized with a BagMixer 400 SW (Interscience, France) and intended for culture. The number of aerobic mesophilic bacteria was determined on nutrient agar plates (BTL, Łódź, Poland) incubated at 30 °C for 24 h. The presence of Gram-negative *Enterobacteriaceae* was determined on Violet, Red, Bile and Glucose (VRBG) agar plates (BioMaxima, Lublin, Poland), after incubation at 37 °C for 24 h. For *E. coli*, Tryptone Bile X-glucuronide (TBX) agar (BioMaxima, Lublin, Poland) was used, and the inoculated media were incubated at 44 °C for 24 h. The number of bacteria was expressed as the number of colony-forming units (CFU) in 1 g of sample.

The second subsample was suspended in 90 mL Selenite-F (SF) broth (BTL, Łódź, Poland) and incubated at 43 °C for 24 h. An inoculation loop full of each SF suspension with sample was streaked onto *Salmonella Shigella* (SS) agar (BTL, Łódź, Poland) and incubated at 37 °C for 24 h. Bacteria, isolated on VRBG, TBX and SS media, were subjected to genera/species identification.

### 2.2. Determination of Bacteria Species via Biochemical and Molecular Methods

#### 2.2.1. Biochemical Tests

Preliminary identification of the isolated strains was carried out using the following sets of commercial kits: ENTEROtest 24N—for *Salmonella*, *E. coli* and other oxidase-negative bacteria from *Enterobacteriaceae*; NEFERMtest 24N—for oxidase-positive non-fermenting bacteria; OXItest—a supplementary test for detecting bacterial cytochrome oxidase; and INDOLtest—for detection of *E. coli* and screening differentiation of indole-positive and indole-negative bacterial genera (Erba-Lachema, Brno, Czech Republic). All tests were performed in accordance with the manufacturers’ recommendations using the ErbaScan absorbance microplate reader with a measurement range from 0.000 to 4.000 OD (Erba-Lachema, Brno, Czech Republic). The interpretation of the ENTEROtest 24N and NEFERMtest 24N results was performed using ErbaExpert microbiological software version 1.2.013 (Erba-Lachema, Brno, Czech Republic). In addition, an analysis of isolated strains was also performed using the Gen III BIOLOG system (BIOLOG Inc., Hayward, CA, USA). The results were read by MicroLog M 5.2 software (BIOLOG Inc., Hayward, CA, USA).

#### 2.2.2. Molecular Tests

Isolation of DNA from bacterial cultures was performed using the Qiamp^®^ DNA Mini Kit (Qiagen, Hilden Germany), according to the protocol for Gram-negative bacteria extraction. One culture loop from a 24-h bacterial culture was taken for isolation. Bacterial DNA was detected by amplification of the 16S rRNA gene fragment using the universal oligonucleotide primers p27f and p1525r according to the method by Chun and Goodfellow [16]. Each reaction has a volume of 50 µL and consisted of 1.5 U Taq DNA Polymerase, 1 × PCR buffer containing 15 mM MgCl2 (Qiagen, Hilden, Germany) and 0.2 mM dNTPs (Thermo Scientific, Waltham, MA, USA), 0.4 µM of each primer (Institute of Biochemistry and Biophysics, Warsaw, Poland) and 5 µL each of DNA template and nuclease-free water (Qiagen, Hilden, Germany). The reaction was conducted on a C1000 Thermal Cycler (BioRad, Hercules, CA, USA). Products of 1500 bp amplification were visualized in 1.5% agarose gel (Prona, Basica LE, Prona, Spain) after electrophoresis in standard conditions and staining with ethidium bromide solution (2 μg/mL).

The PCR sequencing reaction was performed using a BigDye™ Terminator v3.1 Cycle Sequencing Kit (Applied Biosystems, Waltham, MA, USA), and the reaction products were purified using a BigDye XTerminator™ Purification Kit (Applied Biosystems, Waltham, MA, USA). Sequencing was performed on the ABI PRISM 310 Genetic Analyzer (Applied Biosystems, Waltham, MA, USA). The nucleotide sequences were compared with sequences in GenBank using the Basic Local Alignment Search Tool (BLAST).

### 2.3. The Survival of E. coli Present in Organic Fertilizers on a Laboratory Scale

#### 2.3.1. Samples

In the initial phase, the total number of Gram-negative *Enterobacteriaceae* and *E. coli* was determined in the soil (universal soil—used for, e.g., gardening—and clay), sewage sludge and digestate samples used in the experiment (Table 1). The sewage sludge and digestate samples were subjected to preliminary heat treatment at 121 °C for 15 min to remove natural microflora. Sterile samples were intended for inoculation with *E. coli* suspension. No *Salmonella* spp. was detected in any samples.

#### 2.3.2. Inoculum Preparation

The reference strain of *E. coli* ATCC 25922 was used to prepare the inoculum. From the 24-h culture, a suspension was prepared with an optical density of 0.5 McFarland (optical density at 550 nm: 0.125), measured with a Densi-La-Meter II densitometer (Erba-Lachema, Brno, Czech Republic). The initial suspension density (2.05 × 10^8^ CFU/g) was determined based on the average concentration of mesophilic bacteria in the tested non-sterile sewage sludge and digestate samples (Table 2). When the *E. coli* suspension was added to the sterile sewage sludge and digestate samples, the final concentration was 1.8 × 10^6^ CFU/g.

#### 2.3.3. Main Experiment

Four containers were prepared, filled with non-sterile soil (universal or clay) in a volume of 8.3 dm^3^, ensuring the mapping of the top 20 cm of the cultivated surface layer. Two containers were filled with soil, with the addition of 14.2 g and 88.5 g of sterile sewage sludge inoculated with 1.8 mL and 11.1 mL of the stock *E. coli* suspension of the same 2.05 × 10^8^ CFU/g concentration, respectively. The same proportions were used to add digestate samples. The amount of the added sewage sludge or digestate sample was determined based on permissible doses of fertilizers (min. 3 t/ha; max. 20 t/ha) included in the Regulation of the Minister of the Environment [3]. The samples were mixed thoroughly and stored at a temperature of 20–25 °C during the day and 15–20 °C at night. Microbiological contamination was carried out after the 1st, 2nd and 3rd weeks of storage.

#### 2.3.4. Control Group

The control group consisted of universal and clay soil samples with additives of non-sterile sewage sludge and digestate in amounts of 14.2 g and 88.5 g, with the concentration determined in the initial test (Table 1).

## 3. Results

### 3.1. Bacterial Concentration in Soil, Sewage Sludge and Digestate Samples

**Figure 1:**
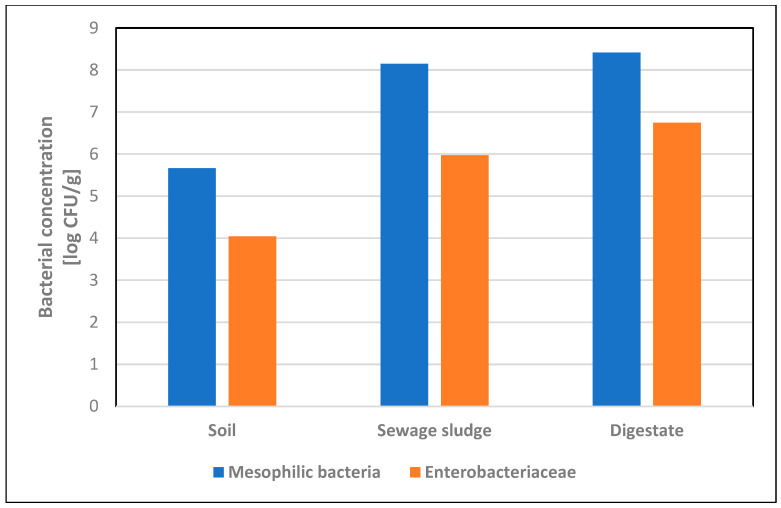
Concentrations [log CFU/g] of mesophilic bacteria and *Enterobacteriaceae* in soil, sewage sludge and digestate samples.

The average concentration of mesophilic bacteria in soil samples was 4.6 × 10^5^ CFU/g, and the average concentration of *Enterobacteriaceae* was 1.1 × 10^4^ CFU/g. *Escherichia coli* was detected in two soil samples, with an average concentration of 25.3 CFU/g. Microbiological analysis of sewage sludge showed the presence of mesophilic bacteria in eight (88.9%) samples, with an average concentration of 1.4 × 10^8^ CFU/g, and *Enterobacteriaceae* in six samples (66.7%), with an average concentration of 9.4 × 10^5^ CFU/g. *Escherichia coli* was detected in four (44.4%) samples, with an average concentration of 1.7 × 10^4^ CFU/g. Microbiological analysis of digestate showed the presence of mesophilic bacteria and *Enterobacteriaceae* in all tested samples, obtaining average concentrations of 2.6 × 10^8^ CFU/g and 5.6 × 10^6^ CFU/g, respectively, while *E. coli* was detected in six (66.7%) samples (Table 2). Among all tested samples, *Enterobacteriaceae* isolated on VRBG medium constituted over 70% of the total number of mesophilic bacteria isolated on nutrient agar (Figure 1). In one digestate sample, the presence of *Salmonella* was confirmed [17].

### 3.2. Species Diversity of Enterobacteriaceae Isolated from Soil, Sewage Sludge and Digestate

From the soil samples, Gram-negative bacteria belonging to the *Serratia* (n = 44), *Enterobacter* (n = 37), *Pantoea* (n = 32), *Citrobacter* (n = 27) and *Pseudomonas* (n = 25) genera were identified the most frequently. Individual cases were confirmed for the genera *Ewingella*, *Gibbsiella*, *Hafnia*, *Kluyvera* and *Yersinia*. In 14 of the 82 samples tested, the presence of *Escherichia coli*, considered one of the main bacterial indicators of soil microbiological purity, was confirmed (Table 3), but in no sample did the concentration exceed the permissible value of 1000 CFU/g. Some species determined by biochemical methods could not be confirmed by sequencing (i.e., *Burkholderia cepacia* complex, *Lelliottia amnigena*, *Chryseobacterium indologenes*, *Methylobacterium mesophilicum*). The bacterial composition in each tested soil sample is provided in the Supplementary Materials (Table S1).

In both sewage sludge and digestate samples, the most frequently identified bacterium was *E. coli*. Some species were detected only in sewage sludge samples (*Alcaligenes faecalis*, *Comamonas jiangduensis*, *Enterobacter cloacae*, *Hafnia alvei*, *Morganella morganii* subsp. *Morganii*), and others only in digestates (*Citrobacter freundii*, *C*. *gillenii*, *Ignatzschineria indica*, *Proteus mirabilis*). The genera *Klebsiella* and *Yersinia* were isolated from both sample types but identified as different species. *Salmonella enterica* subsp. enterica (serotype Johannesburg) was identified in only one digestate sample (Table 4). Of all species identified via biochemical methods, *Raoultella terrigena*, *Brevundimonas diminuta* and *Oligella urethralis* were not confirmed by sequencing (Table S2) [18].

**Table 3:** *Enterobacteriaceae* isolated from the soil samples.

Enterobacteriaceae Isolated from Soil Samples (n = 82)		Sequence Result (Similarity with 16S rRNA)		Impact on Human Health		
*Aeromonas* spp. (n = 13)	*A. hydrophila*	100%	MT572504.1	opportunistic pathogen	emerging foodborne pathogen capable of causing human gastroenteritis	[19]
	*A. sobria*	99.79%	OL778934.1		food-borne illness, severe extraintestinal diseases: sepsis, peritonitis, meningitis	[20]
	*A. media*	99.82%	CP038448.1		food-borne illness, diarrhea	[21]
	*A. veronii*	99.41%	KF853564.1		gastroenteritis, bacteremia, septicemia, wound infections	[22]
	*A. salmonicida*	99.54%	MT576565.1		strain isolated from the blood of the patient with endocarditis	[23]
	*A. encheleia*	100%	MT436428.1		-	-
*Achromobacter* spp. (n = 2)	*A. xylosoxidans* subsp. *xylosoxidans*	99.77%	CP054571.1	opportunistic pathogen	bacteremia, meningitis, urinary tract infection, endocarditis, pneumonia, especially in immunocompromised patients	[24]
*Buttiauxella* spp. (n = 4)	*B. agrestis*	99.57%	AP023184.1	rare opportunistic pathogen	*B. agrestis* infection occurring at a post-cesarean surgical site	[25]
	*B. gaviniae*	100%	MK905440.1	-	clinical strain isolated from a urine sample from a spinal cord patient with urinary bladder pathology	[26]
*Citrobacter* spp. (n = 27)	*C. braakii*	99.57%	MT534007.1	rare pathogen, nosocomial infections	bacteremia in immunocompromised patients	[27,28]
	*C. freundii*	100%	MH045703.1	opportunistic pathogen of a wide spectrum of nosocomial infections	food poisoning, diarrhea, urinary tract infections	[29,30]
*Enterobacter* spp. (n = 37)	*E. cloacae* subsp. *cloacae*	99.83%	MN181145.1	opportunistic pathogen, nosocomial infections	lower respiratory tract infections, bacteremia	[31]
	*E. ludwigii*	99.71%	MN826154.1	unusual human pathogen	nosocomial bloodstream infection	[32]
	*E*. *asburiae*	99.67%	MN709316.1	opportunistic pathogen	nosocomial infections	[33]
	*E. amnigenus* biovar 1	100%	MN658356.1	unusual human pathogen	nosocomial infections	[34]
*Escherichia* spp. (n = 14)	*E. coli*	100%	MT192520.1	opportunistic pathogen	enteritis, urinary tract infection, septicemia, neonatal meningitis, diarrhea	[35]
	*E. vulneris*	99.57%	KX357823.1	possible opportunistic pathogen	wound infections; complicated diarrhea and sepsis in an infant	[36,37]
*Ewingella* spp. (n = 1)	*E. americana*	100%	MT998223.1	rare opportunistic pathogen	bacteremia, pneumonia, conjunctivitis, Waterhouse–Friderichsen syndrome, peritonitis	[38]
*Gibbsiella* spp. (n = 1)	*G. quercinecans*	99.78%	MN822736.1	-	-	-
*Hafnia* spp. (n = 2)	*H. alvei*	99.78%	CP050150.1	potentially opportunistic pathogen	rare pneumonia cases	[39,40]
*Klebsiella* spp. (12)	*K. pneumoniae* subsp. *pneumoniae*	99.79%	OM017200.1	opportunistic pathogen	urinary and respiratory tract infections, liver abscess, endophthalmitis, meningitis	[41]
	*K. oxytoca*	100%	MT568561.1	opportunistic pathogen	colitis, infective endocarditis, urinary and respiratory tract infections associated with nosocomial infections	[42]
*Kluyvera* spp. (n = 2)	*K. intermedia*	99.78%	LT899978.1	potential opportunistic pathogen	soft tissue infections, urinary tract infections, intra-abdominal abscesses, catheter-associated bloodstream infections, septic shock in immunocompromised patients (nosocomial infections)	[43]
*Pantoea* spp. (n = 32)	*P. agglomerans*	99.76%	MT635441.1	opportunistic pathogen	septic arthritis, synovitis endophthalmitis, periostitis, endocarditis and osteomyelitis in the event of wound infection with plant material or as a hospital-acquired infection, mostly in immunocompromised individuals	[44]
*Pseudomonas* spp. (n = 25)	*P. tolaasii*	99.56%	MT561438.1	-	-	-
	*P. abietaniphila*	100%	MH379754.1	-	-	-
	*P. fluroescens*	99.73%	OM827287.1	scarce clinical significance	nosocomial infections	[45]
	*P. koreensis*	100%	MT501807.1	-	possible nosocomial infections; a case of contact lens-related mixed infectious keratitis caused by *A. fumigatus* and *P. koreensis*	[46,47]
	*P. brassicacearum* subsp. *neoaurantiaca*	99.80	MT634587.1	-	-	-
	*P. chlororaphis*	100%	KJ530973.1	-	-	-
	*P. kilonensis*	99.57%	MT102732.1	-	-	-
*Rahnella* spp. (n = 18)	*R. aquatilis*	99.77%	MN826573.1	possible opportunistic pathogen	possible role as the pathogen responsible for ventilator-associated pneumonia associated with nosocomial infections in immunocompromised patients	[48]
	*R. victoriana*	99.78%	OK658118.1	-	-	-
*Raoultella* spp. (n = 5)	*R. terrigena*	100%	MT545123.1	opportunistic pathogen	cases of bloodstream, urinary tract, respiratory tract or bile tract infections mostly associated with nosocomial infections; subungual abscess caused by *R. terrigena*	[49,50]
	*R. ornithinolytica*	100%	MT568560.1			
*Serratia* spp. (n = 44)	*S. plymuthica*	99.58%	CP053398.1	rare (unusual) human pathogen	associated with chronic osteomyelitis and cases of sepsis secondary to central venous catheter infection	[51]
	*S. liquefaciens*	100%	MT279350.1	opportunistic pathogen	cause of transfusion-related sepsis, meningitis thrombophlebitis, corneal ulcers	[52]
	*S. fonticola*	100%	MN227497.1	rare (unusual) human pathogen	skin and soft tissue infections	[53,54]
	*S. quinivorans*	99.71%	MT256279.1	-	-	-
	*S. proteamaculans*	100%	MK530287.1	opportunistic pathogen	able to penetrate eukaryotic cells	[55]
	*S. marcescens*	99.85%	MT598027.1	opportunistic pathogen	urinary tract infections, pneumonia, intravenous catheter-associated infections, osteomyelitis, endocarditis	[51,56]
	*S. entomophila*	99.83%	MK216954.1	-	-	-
*Yersinia* spp. (n = 2)	*Y. enterocolitica*	99.14%	MN905014.1	zoonotic pathogen	enteric infections, mesenteric lymphadenitis, reactive arthritis, erythema nodosum	[57]

**Table 4:** *Enterobacteriaceae* isolated from the sewage sludge and digestate samples.

Enterobacteriaceae Isolated from the Sewage Sludge and Digestate Samples (n = 18)	Sequence Result (Similarity with 16S rRNA)				Impact on Human Health	
	Sewage Sludge Samples		Digestate Samples			
*Aeromonas salmonicida*	100%	MT576565.1	100%	KF551980.1	As in Table 2	
*Alcaligenes faecalis*	99.76%	MT277037.1	-	-	sporadic cases of endocarditis, meningitis, chronic otitis, pyelonephritis, bacteremia, peritonitis, endophthalmitis, abscesses, often associated with nosocomial infections	[58]
*Comamonas jiangduensis*	99.80%	NR_109655.1	-	-	-	-
*Citrobacter freundii*	-	-	100%	OM666544.1	As in Table 2	
*Citrobacter gillenii*	-	-	100%	MT436425.1	the strains were isolated from human stool, urine and blood	[59]
*Enterobacter cloacae*	99.61%	MN006380.1	-	-	As in Table 2	
*Escherichia coli*	100%	OM982954.1	100%	CP091756.1	As in Table 2	
*Hafnia alvei*	99.39%	CP050150.1	-	-	As in Table 2	
*Ignatzschineria indica*			99.75%	LC010924.1	rare cases of bacteremia mostly associated with wound myiasis	[60]
*Klebsiella oxytoca*	99.48%	MT509911.1	-	-	As in Table 2	
*Klebsiella pneumoniae*	-	-	99.79%	OM978275.1	As in Table 2	
*Morganella morganii* subsp. *morganii*	99.80%	CP043955.1	-	-	opportunistic infections: urinary tract infection, wound infection, arthritis, prostatitis	[61]
*Proteus mirabilis*	-	-	99.63%	OM882519.1	catheter-associated urinary tract infections	[62]
*Salmonella enterica* subsp. *enterica* serovar Johannesburg	-	-	100%	CP049308.1	gastrointestinal infection—salmonellosis	[63]
*Yersinia enterocolitica*	-	-	99.71%	MK910030.1	As in Table 2	
*Yersinia frederiksenii*	100%	KC776774.1	-	-	possible diarrheal diseases	[64]
*Yersinia intermedia*	-	-	99.82%	MN416246.1	strains were isolated from human stool and urine samples and from wound infections but rarely associated with human diseases	[65]

### 3.3. The Survival of E. coli in Soil Samples Fertilized with Sewage Sludge and Digestate in Laboratory Conditions

#### 3.3.1. Main Experiment

After the first week of fertilization with sewage sludge, an increase in the total number of *Enterobacteriaceae* was found compared to the initial result found in unfertilized soil (3.1 × 10^2^ CFU/g): up to 9.7 × 10^3^ CFU/g and 2.9 × 10^5^ CFU/g for universal and clay soils, respectively. In the case of applying the minimum dose of sewage sludge, after the third week, the values did not exceed 100 CFU/g, while at the maximum dose, the final results were similar to the results of the unfertilized soil (1.3 × 10^2^ and 6.0 × 10^2^ CFU/g). The highest *E. coli* concentration values were obtained after the first week: up to 5.7 × 10^3^ CFU/g and 1.8 × 10^5^ CFU/g for universal and clay soils, respectively. After three weeks, the results decreased below 1 CFU/g, except for the application of the maximum dose of fertilizer in clay soil (2.8 × 10^2^ CFU/g).

When digestate was used in all four variants, *E. coli* concentrations dropped to <1 or 16 CFU/g after three weeks. In the case of *Enterobacteriaceae*, after using the minimum dose of digestate, the bacterial concentration did not exceed 70 CFU/g. At the maximum dose, the final values were similar to those for unfertilized soil and amounted to an average of 2 × 10^2^ CFU/g for both types of soil (Table 5).

#### 3.3.2. Control Experiment

In both types of soil with the addition of non-sterile sewage sludge or digestate, no growth of *E. coli* bacteria was observed. In universal soil with sewage sludge and clay soil with digestate at the maximum dose, the final concentration of *Enterobacteriaceae* obtained after three weeks was lower (<1.8 × 10^2^ CFU/g) than the initial value for unfertilized soil (3.1 × 10^2^ CFU/g). In the remaining variants, the final concentration of *Enterobacteriaceae* did not exceed 100 CFU/g (Table 6).

## 4. Discussion

The use of sewage sludge and digestate as fertilizer on arable land promotes the functioning of the soil ecosystem, increasing crop productivity. In this study, high levels of contamination were found in sewage sludges and digestate: 1.4 × 10^8^ CFU/g and 2.6 × 10^8^ CFU/g, respectively. Slightly lower degrees of organic fertilizer contamination with mesophilic bacteria have been recorded in Spain (2.4 × 10^7^ CFU/g for sludge) [66] and Germany (0.5 × 10^6^ CFU/g for digestate) [67]. Direct sewage or digestate introduction into soil could increase the risk of environmental exposure to microbiological contamination, thus posing a threat to human and animal health. As indicated in Table 3 and Table 4, bacteria posing no direct health risk and those with pathogenic properties were isolated from the tested organic substances.

Currently, it is not possible for every organic sample introduced as fertilizer to be tested for bacterial species, including the quantitative assessment of individual species. Most legislation regulating the biological safety of fertilizers in terms of bacteria is based on tests for the detection of *Salmonella*. Of the samples tested, only one digestate sample confirmed the presence of *Salmonella* (*S*. *enterica* subsp. *enterica* serovar Johannesburg), which excludes the possibility of its introduction into the soil for agricultural purposes. In other studies, the presence of *Salmonella* spp. in digestates varies depending on the origin of the material and ranges from 8% (1/12) [68] to 100% (5/5) [69]. The presence of *Salmonella* spp. in sewage sludges is recorded at the level of 26.7% (4/15) [70] or 38.9% (21/54) [71].

The second frequently used indicator of microbiological contamination of soils and fertilizers is *E. coli*, which is a representative species of the *Enterobacteriaceae* family. The current study showed a higher average number of *E. coli* in the digestate samples, amounting to 1.7 × 10^6^ CFU/g, than in the sewage sludge samples, which were determined to be 1.7 × 10^4^ CFU/g. Studies on raw digestates collected from biogas plants in France showed mean *E. coli* counts ranging from 9.4 × 10^1^ to 1.3 × 10^4^ CFU/g [69]. The presence of *E. coli* strains in sewage sludge may suggest that treatment methods are not effective and that bacteria may be introduced into the soil environment, including pathogenic strains. The average counts of these bacterial strains may vary depending on the origin of the sewage sludge. Korzeniewska et al. [72] showed that the mean number of *E. coli* bacteria in untreated hospital sewage ranged from 6 × 10^2^ to 1 × 10^5^ CFU/mL, whereas in municipal sewage, it was higher and ranged from 1.1 × 10^3^ to 1.3 × 10^5^ CFU/mL. In a screening study in Sweden, the mean number of *E. coli* also varied depending on the sampling period in the effluent and ranged from 5.0 × 10^1^ to 9.15 × 10^2^ CFU/mL [73].

The results from our laboratory experiments revealed the presence of *Enterobacteriaceae* and *E. coli* at the levels below 1000 CFU/g recorded within 2 weeks of fertilization. After the first week, an increase in the number of *E. coli* specimens was noted in the universal soil sample. Similar results were obtained in the study conducted by Qiao et al. [74], who also showed an increase in the concentration of *E. coli* bacteria after 8 days. However, in the experiment conducted on clayey soil, the opposite results were observed, characterized by a decrease in the concentration of these microorganisms after a week. Moreover, higher concentrations of the tested microorganisms persisted longer in clay soil compared to sandy soil. The obtained results are consistent with those obtained by Alegbeleye and Sant’Ana [75], who also confirmed the higher survival of *E. coli* strains in clay soil compared to sandy soil. The persistent microbiological contamination of fertilized soil also depends on the dose of fertilizer applied. The use of smaller doses of fertilizers significantly accelerates the reduction in potentially pathogenic bacteria in the fertilized soil, and thus increases the safety of people in contact with it. However, further research in this area under natural conditions using experimental plots is necessary.

Taking into account the obtained diversity of the microbiome, the assessment of the microbiological purity of sediments and fermentates, referring mainly to *E. coli* and *Salmonella* spp., does not fully demonstrate the potential risk resulting from human exposure to pathogens. High levels of contamination based on the total number of microorganisms may indicate a need to improve sanitation methods used in biogas plants and sewage treatment plants. Despite these difficulties, sewage sludge and digestate are being increasingly used in agriculture as rich sources of plant nutrients and because the beneficial chemical elements they contain, including nitrogen, decompose slowly, providing nutrients over an extended period of time. Additionally, their use as fertilizers is an alternative to conventional waste disposal.

However, it is also important to appropriately adapt existing regulations regarding limiting potential human contact with pathogens [76,77]. Experimental field studies conducted in Poland on the effect of regular use of sewage sludge to fertilize agricultural soils showed significant quantitative and qualitative changes in the composition of the soil microbiota, disturbing its balance and influencing the processes occurring within it [75].

## 5. Conclusions

Based on the results of the experiments conducted in laboratory conditions, it can be concluded that adding the minimum dose (corresponding to a value of 3 t/ha) of sewage sludge or digestate with *Enterobacteriaceae* contamination below 2.5 × 10^6^ CFU/g to soil, after an initial increase in the concentration of bacteria, does not result in bacterial concentrations exceeding the permissible value of 1000 CFU/g after weeks. Similarly, if the maximum dose (corresponding to a value of 20 t/ha) is used, the final results are at the same level found in unfertilized soil (2 × 10^2^ CFU/g). Considering the species composition of soil, sewage sludge and digestate, including both pathogenic and non-pathogenic microorganisms, it is justified to change existing regulations by abolishing the obligation to quantitatively test samples for the presence of bacteria from the *Enterobacteriaceae* family. However, further research under natural conditions is necessary to confirm the biological safe use of sewage sludge and digestate as fertilizers.

## Figures and Tables

**Table 1:** Initial bacterial contamination of soil, sewage sludge and digestate samples (before heat treatment).

Component	Total No. of *Enterobacteriaceae* [CFU/g]	Total No. of *Escherichia coli* [CFU/g]
Universal soil	3.1 × 10^2^	<1
Clay soil	26	<1
Sewage sludge	2.5 × 10^6^	1.4 × 10^4^
Digestate	1.3 × 10^6^	1.0 × 10^4^

**Table 2:** Concentration of bacteria in soil, sewage sludge and digestate samples.

Type of Samples	Bacterial Concentration [CFU/g]								
	Total Mesophilic Bacteria			*Enterobacteriaceae*			*Escherichia coli*		
	Positive Samples	Median (Range)	Mean ± S.D.	Positive Samples	Median (Range)	Mean ± S.D.	Positive Samples	Median (Range)	Mean ± S.D.
Soil n = 82	82 (100%)	3.8 × 10^5^ (1.1 × 10^5^−4.6 × 10^6^)	4.6 × 10^5^ ± 5.1 × 10^5^	82 (100%)	6.3 × 10^2^ (1.2 × 10^1^−3.2 × 10^5^)	1.1 × 10^4^ ± 4.3 × 10^4^	2 (2.4%)	−	−
Sewage sludge n = 9	8 (88.9%)	3.7 × 10^7^ (1.6 × 10^3^−5.7 × 10^8^)	1.4 × 10^8^ ± 2.1 × 10^8^	6 (66.7%)	7.2 × 10^5^ (1.5 × 10^3^−2.2 × 10^6^)	9.4 × 10^5^ ± 9.9 × 10^5^	4 (44.4%)	1.2 × 10^4^ (3.8 × 10^2^−4.2 × 10^4^)	1.7 × 10^4^ ± 2.0 × 10^4^
Digestate n = 9	9 (100%)	1.2 × 10^8^ (1.0 × 10^7^−9.8 × 10^8^)	2.6 × 10^8^ ± 3.4 × 10^8^	9 (100%)	2.8 × 10^6^ (3.1× 10^5^−2.1 × 10^7^)	5.6 × 10^6^ ± 7.3 × 10^6^	6 (66.7%)	3.1 × 10^4^ (8.0 × 10^1^−9.9 × 10^6^)	1.7 × 10^6^ ± 4.0 × 10^6^

**Table 5:** Concentration of *Enterobacteriaceae* in soil samples fertilized with sterile sewage sludge/digestate inoculated with an *E. coli* suspension.

Type of Sample: Non-Sterile Soil + Sterile Sewage Sludge/Digestate + *E. coli* Suspension	Week	*Enterobacteriaceae* [CFU/g]	*E*. *coli* [CFU/g]
universal soil + sewage sludge + *E. coli* (8.3 dm^3^ + 14.2 g + 1.8 mL)	0	3.3 × 10^3^	1.1 × 10^3^
	1	1.3 × 10^3^	5.7 × 10^3^
	2	1.8 × 10^2^	<1
	3	41	<1
universal soil + sewage sludge + *E. coli* (8.3 dm^3^ + 88.5 g + 11.1 mL)	0	1.2 × 10^4^	1.6 × 10^3^
	1	9.7 × 10^3^	3.5 × 10^3^
	2	8.0 × 10^2^	2.4 × 10^3^
	3	1.3 × 10^2^	<1
universal soil + digestate + *E. coli* (8.3 dm^3^ + 14.2 g + 1.8 mL)	0	1.4 × 10^3^	0.9 × 10^2^
	1	3.2 × 10^3^	1.0 × 10^2^
	2	2.3 × 10^2^	<1
	3	67	<1
universal soil + digestate + *E. coli* (8.3 dm^3^ + 88.5 g + 11.1 mL)	0	3.0 × 10^3^	3.9 × 10^3^
	1	6.4 × 10^3^	4.0 × 10^3^
	2	1.8 × 10^2^	1.0 × 10^2^
	3	1.8 × 10^2^	<1
clay soil + sewage sludge + *E. coli* (8.3 dm^3^ + 14.2 g + 1.8 mL)	0	2.2 × 10^3^	1.8 × 10^3^
	1	2.4 × 10^2^	1.3 × 10^2^
	2	1.2 × 10^2^	21
	3	83	<1
clay soil + sewage sludge + *E. coli* (8.3 dm^3^ + 88.5 g + 11.1 mL)	0	1.0 × 10^4^	1.3 × 10^4^
	1	2.9 × 10^5^	1.8 × 10^5^
	2	1.6 × 10^4^	5.3 × 10^3^
	3	6.0 × 10^2^	2.8 × 10^2^
clay soil + digestate + *E. coli* (8.3 dm^3^ + 14.2 g + 1.8 mL)	0	1.8 × 10^3^	3.1 × 10^3^
	1	1.9 × 10^2^	1.6 × 10^2^
	2	41	13
	3	15	<1
clay soil + digestate + *E. coli* (8.3 dm^3^ + 88.5 g + 11.1 mL)	0	1.6 × 10^4^	1.5 × 10^4^
	1	1.8 × 10^3^	1.3 × 10^3^
	2	2.1 × 10^2^	25
	3	2.2 × 10^2^	16

**Table 6:** Concentration of *Enterobacteriaceae* in the soil samples fertilized with non-sterile sewage sludge and digestate.

Type of Sample: Non-Sterile Soil + Non-Sterile Sewage Sludge/Digestate	Week	*Enterobacteriaceae* [CFU/g]	*E*. *coli* [CFU/g]
universal soil + sewage sludge (8.3 dm^3^ + 14.2 g)	0	1.3 × 10^4^	<1
	1	3.5 × 10^2^	<1
	2	1.2 × 10^2^	<1
	3	1.1 × 10^2^	<1
universal soil + sewage sludge (8.3 dm^3^ + 88.5 g)	0	3.5 × 10^3^	2.5 × 10^2^
	1	9.5 × 10^2^	65
	2	1.2 × 10^2^	<1
	3	1.7 × 10^2^	<1
universal soil + digestate (8.3 dm^3^ + 14.2 g)	0	2.3 × 10^3^	<1
	1	3.3 × 10^2^	<1
	2	37	<1
	3	84	<1
universal soil + digestate (8.3 dm^3^ + 88.5 g)	0	1.3 × 10^3^	<1
	1	4.6 × 10^2^	<1
	2	57	<1
	3	97	<1
clay soil + sewage sludge (8.3 dm^3^ + 14.2 g)	0	95	<1
	1	1.3 × 10^3^	<1
	2	2.8 × 10^2^	<1
	3	32	<1
clay soil + sewage sludge (8.3 dm^3^ + 88.5 g)	0	117	<1
	1	9.3 × 10^3^	<1
	2	3.0 × 10^2^	<1
	3	55	<1
clay soil + digestate (8.3 dm^3^ + 14.2 g)	0	49	<1
	1	1.1 × 10^2^	<1
	2	11	<1
	3	15	<1
clay soil + digestate (8.3 dm^3^ + 88.5 g)	0	1.3 × 10^2^	<1
	1	1.5 × 10^3^	<1
	2	1.4 × 10^2^	<1
	3	1.8 × 10^2^	<1

## Data Availability

All data generated or analyzed during this study are included in this published article and Supplementary Materials, and they are available from the Department of Health Biohazards and Parasitology (Institute of Rural Health, Lublin, Poland).

## Supplementary Materials

The following supporting information can be downloaded at: https://www.mdpi.com/article/10.3390/pathogens13121056/s1, Table S1: Results of quantitative and qualitative analysis of bacteriological contamination of soil samples; Table S2: Results of quantitative and qualitative analysis of bacteriological contamination of sewage sludge and digestate samples.
