# Supplementary material for: Enterobacteriaceae in Sewage Sludge and Digestate Intended for Soil Fertilization
Source: Pathogens. 2024 Nov 30;13(12):1056. doi: 10.3390/pathogens13121056 (PMC11728692; doi:10.3390/pathogens13121056)
Supplement: Supplementary file 1 [file pathogens-13-01056-s001.zip › pathogens-3295405-supplementary.pdf]

Supplementary materials

Table S1. Results of quantitative and qualitative analysis of bacteriological contamination of soil samples

**No. of sample**	**Bacterial concentration**			**Presence of *Salmonella***	**Identified species of gram-negative bacteria using biochemical methods**
	**Total mesophilic bacteria [×10^5 CFU/g]**	***Enterobacteriaceae* [×10^3 CFU/g]**	***Escherichia coli* [CFU/g]**		
1	4,2	0,44	<1	nd*	*Serratia* spp.<br>*Klebsiella pneumoniae* subsp. *pneumoniae*
2	2,1	0,99	<1	nd	*Rahnella aquatilis*<br>*Serratia plymuthica*<br>*Klebsiella pneumoniae* subsp. *pneumoniae*
3	4,1	0,74	<1	nd	*Pantoea agglomerans*<br>*Enterobacter* spp.<br>*Escherichia coli*
4	2,8	1,4	<1	nd	*Klebsiella pneumoniae* subsp. *pneumoniae*<br>*Pseudomonas* spp.
5	2,1	1,2	<1	nd	*Pantoea agglomerans*<br>*Serratia plymuthica*
6	1,7	0,26	<1	nd	*Pantoea agglomerans*<br>*Achromobacter xylosoxidans* subsp. *xylosoxidans*

7	2,3	6,6	<1	nd	*Rahnella aquatilis*<br>*Serratia plymuthica*<br>*Enterobacter amnigenus biovar 1*
8	1,6	1,3	<1	nd	*Serratia plymuthica, S. liquefaciens*<br>*Enterobacter cloacae* subsp. *cloacae*
9	3,6	17	<1	nd	*Raoultella terrigena*<br>*Pantoea agglomerans*
10	4,2	22	<1	nd	*Aeromonas* spp., *A. hydrophila* subsp. *hydrophila*<br>*Pantoea agglomerans*
11	3,3	0,41	<1	nd	*Citrobacter braakii*<br>*Pantoea agglomerans*<br>*Enterobacter* spp.
12	3,1	0,43	<1	nd	*Serratia liquefaciens*
13	1,6	0,31	<1	nd	*Serratia* spp.<br>*Citrobacter freundii*
14	1,7	0,15	<1	nd	*Citrobacter braakii*<br>*Serratia* spp., *S. plymuthuca*<br>*Pseudomonas* spp.
15	6,3	1,2	<1	nd	*Citrobacter braakii*<br>*Burkholderia cepacia complex*

16	4,1	8	<1	nd	*Enterobacter* spp.<br>*Pantoea agglomerans*<br>*Burkholderia cepacia* complex
17	2,8	1,1	<1	nd	*Pantoea agglomerans*<br>*Klebsiella pneumoniae* subsp. *pneumoniae*<br>*Serratia* spp.
18	5,2	0,78	<1	nd	*Pantoea agglomerans*<br>*Serratia proteamaculans*<br>*Citrobacter freundii*<br>*Enterobacter* spp.<br>*Burkholderia cepacia* complex
19	4,3	0,17	<1	nd	*Pantoea agglomerans*<br>*Enterobacter* spp.<br>*Serratia* spp.<br>*Burkholderia cepacia* complex
20	9,7	0,31	<1	nd	*Pantoea agglomerans*<br>*Enterobacter* spp., *E. cloacae* subsp. *cloacae*<br>*Escherichia coli*<br>*Burkholderia cepacia* complex
21	2	0,63	<1	nd	*Pantoea agglomerans*<br>*Enterobacter* spp.

					Escherichia coli<br>*Burkholderia cepacia* complex
22	3,2	0,29	<1	nd	*Pantoea agglomerans*<br>*Enterobacter* spp.<br>*Serratia* spp.<br>*Burkholderia cepacia* complex
23	2,3	0,41	<1	nd	*Lelliottia amnigena*<br>*Citrobacter freundii*<br>*Serratia* spp.<br>*Escherichia coli*<br>*Achromobacter xylosoxidans* subsp. *xylosoxidans*<br>*Burkholderia cepacia* complex
24	1,6	0,31	<1	nd	*Pantoea agglomerans*<br>*Enterobacter* spp., *E. cloacae* subsp. *cloacae*<br>*Serratia* spp.
25	2,7	0,57	<1	nd	*Citrobacter braakii*<br>*Enterobacter* spp.<br>*Serratia* spp.<br>*Escherichia coli*<br>*Burkholderia cepacia* complex

26	1,3	0,52	<1	nd	*Pantoea agglomerans*<br>*Enterobacter* spp.<br>*Serratia liquefaciens*<br>*Pseudomonas tolaasii*<br>*Burkholderia cepacia* complex
27	3,2	0,88	<1	nd	*Raoultella ornithinolytica*<br>*Enterobacter cloacae* subsp. *cloacae*<br>*Escherichia coli*<br>*Burkholderia cepacia* complex
28	4,1	2,5	<1	nd	*Pantoea agglomerans*<br>*Rahnella aquatilis*<br>*Klebsiella pneumoniae* subsp. *pneumoniae*<br>*Serratia* spp.<br>*Burkholderia cepacia* complex
29	4,9	0,3	<1	nd	*Pantoea agglomerans*<br>*Burkholderia cepacia* complex
30	5,9	1,7	<1	nd	*Escherichia coli*<br>*Pantoea agglomerans*<br>*Pseudomonas* spp.
31	10	22	<1	nd	*Klebsiella pneumoniae* subsp. *pneumoniae*<br>*Pantoea agglomerans*

					Rahnella aquatilis<br>*Enterobacter amnigenus biovar 1*
32	3,9	0,41	<1	nd	*Klebsiella pneumoniae* subsp. *pneumoniae*<br>*Serratia liquefaciens*<br>*Citrobacter freundii*<br>*Pseudomonas* spp.<br>*Pantoea agglomerans*<br>*Rahnella aquatilis*
33	7,5	8,1	<1	nd	*Enterobacter cloacae*<br>*Citrobacter freundii*<br>*Pantoea agglomerans*
34	3,4	0,88	<1	nd	*Pantoea agglomerans*<br>*Buttiauxella* spp.
35	5,7	0,54	<1	nd	*Pseudomonas* spp.<br>*Pantoea agglomerans*
36	4,9	5,8	<1	nd	*Citrobacter braakii, C. freundii*<br>*Enterobacter amnigenus biovar 1*<br>*Pantoea agglomerans*
37	2,8	0,13	<1	nd	*Rahnella aquatilis*<br>*Serratia plymuthica*

38	3,2	0,71	<1	nd	*Serratia fonticola*
39	7,3	0,34	<1	nd	*Pantoea agglomerans*<br>*Rahnella aquatilis*
40	3,8	0,54	<1	nd	*Escherichia coli*<br>*Rahnella aquatilis*
41	1,9	0,05	<1	nd	not detected
42	6,2	8,3	<1	nd	*Escherichia vulneris*
43	45,5	3,6	<1	nd	*Enterobacter* spp., *E. cloacae*<br>*Aeromonas encheleia*<br>*Pseudomonas abietaniphila*
44	4,6	4,8	<1	nd	*Enterobacter* spp., *E. amnigenus biovar 1, E. cloacae*<br>*Escherichia vulneris*<br>*Citrobacter braakii*<br>*Pseudomonas* spp.<br>*Aeromonas* spp.
45	3	0,7	<1	nd	*Serratia* spp.<br>*Enterobacter* spp., *E. asburiae*<br>*Klebsiella pneumoniae* subsp. *pneumoniae*

46	4,3	0,042	<1	nd	*Serratia liquefaciens, S. proteamaculans*<br>*Klebsiella pneumoniae* subsp. *pneumoniae*<br>*Enterobacter cloacae* subsp. *cloacae*<br>*Pseudomonas fluorescens, P. koreensis*
47	3,2	0,39	<1	nd	*Serratia gillenii, S. liquefaciens*<br>*Enterobacter* spp.<br>*Citrobacter spp.*<br>*Aeromonas hydrophila* subsp. *hydrophila*<br>*Pseudomonas* spp.
48	4,3	1,4	<1	nd	*Serratia fonticola*
49	7,8	12	<1	nd	*Aeromonas hyrrophila, A. sorbia, media*<br>*Pseudomonas* spp.
50	9,7	0,031	<1	nd	*Lelliottia amnigena*<br>*Serratia liquefaciens*<br>*Enterobacter cloacae*<br>*Citrobacter freundii*<br>*Pseudomonas* spp.<br>*Aeromonas hydrophila*
51	3,4	0,73	<1	nd	*Citrobacter braakii*<br>*Serratia* spp.<br>*Aeromonas hydrophila* subsp. *hydrophila*

					Chryseobacterium indologenes
52	7,5	0,048	<1	nd	*Escherichia vulneris*<br>*Enterobacter* spp.<br>*Pseudomonas brassicacearum subsp. neoaurantiaca*<br>*Methylobacterium mesophilicum*
53	4	1,8	<1	nd	*Serratia quinivorans, S. plymuthica*<br>*Pseudomonas* spp., *P. chlororaphis*<br>*Rahnella aqutilis*
54	2,9	0,056	<1	nd	*Pseudomonas kilonensis*<br>*Pantoea agglomerans*
55	4,5	0,72	<1	nd	*Serratia liquefaciens*<br>*Citrobacter braakii*<br>*Aeromonas media*
56	1,7	0,1	<1	nd	*Serratia plymuthica*<br>*Pseudomonas* spp.<br>*Aeromonas hydrophila* subsp. *hydrophila*
57	3,1	0,63	<1	nd	*Citrobacter* spp., *C. braakii*<br>*Aeromonas* spp., *A. hydrophila* subsp. *hydrophila*

58	1,4	3,3	<1	nd	*Kluyvera intermedia*<br>*Pantoea agglomerans*<br>*Pseudomonas* spp.
59	2,1	0,45	<1	nd	*Rahnella aquatilis*
60	1,1	320	10,5	nd	*Rahnella aquatilis*<br>*Ewingella americana*<br>*Raoultella terrigena, R. ornithinolytica*<br>*Escherichia coli*<br>*Enterobacter* spp.<br>*Klebsiella oxytoca, K. pneumoniae* subsp. *pneumoniae*<br>*Citrobacter braakii*
61	6,2	0,037	<1	nd	*Serratia proteamaculans*<br>*Aeromonas veronii*<br>*Pseudomonas* spp.
62	7,8	1	<1	nd	*Serratia liquefaciens*<br>*Escherichia coli*<br>*Rahnella aquatilis*<br>*Enterobacter amnigenus biovar 1*<br>*Citrobacter* spp.

63	1,8	0,052	<1	nd	*Pantoea agglomerans*<br>*Buttiauxella agrestis*<br>*Citrobacter braakii, C. freundii*<br>*Enterobacter ludwigii*<br>*Pseudomonas* spp.
64	3,8	0,5	<1	nd	*Serratia liquefaciens*<br>*Enterobacter* spp.<br>*Pseudomonas* spp.
65	6,4	0,037	<1	nd	*Serratia* spp., *S. plymuthica*<br>*Enterobacter* spp.<br>*Raoultella terrigena*<br>*Citrobacter* spp.<br>*Pseudomonas* spp.
66	3,8	7,5	40,0	nd	*Enterobacter amnigenus biovar 1*<br>*Raoultella terrigena*<br>*Rahnella victoriana*<br>*Citrobacter braakii*<br>*Yersinia* spp.<br>*Pseudomonas* spp.
67	4,3	230	<1	nd	*Escherichia coli*<br>*Citrobacter* spp.

					Serratia spp.<br>*Yersinia enterocolitica*
68	7,9	1,3	<1	nd	*Serratia fonticola*<br>*Enterobacter* spp.<br>*Pseudomonas* spp.
69	7,5	0,086	<1	nd	*Enterobacter amnigenus biovar 1*<br>*Serratia liquefaciens, S. plymuthica*<br>*Citrobacter braakii*<br>*Pseudomonas* spp.
70	7,6	30	<1	nd	*Rahnella aquatilis*<br>*Serratia marcescens*<br>*Citrobacter* spp., *C. braakii*
71	4,4	2,2	<1	nd	*Rahnella aquatilis*<br>*Klebsiella pneumoniae* subsp. *pneumoniae*<br>*Aeromonas salmonicida*
72	4,3	0,095	<1	nd	*Rahnella aquatilis*<br>*Klebsiella pneumoniae* subsp. *pneumoniae, K. oxytoca*<br>*Serratia quinivorans*<br>*Enterobacter* spp.

73	1,3	0,028	<1	nd	*Rahnella aquatilis*<br>*Serratia liquefaciens*<br>*Citrobacter freundii*<br>*Kluyvera intermedia*<br>*Enterobacter* spp., *E. cloacae*
74	2,3	0,56	<1	nd	*Serratia liquefaciens, S. plymuthica*<br>*Enterobacter* spp.<br>*Pseudomonas* spp.
75	2,7	0,027	<1	nd	*Buttiauxella gaviniae*<br>*Serratia plymuthica, S. liquefaciens*
76	4,5	17	<1	nd	*Buttiauxella agrestis*<br>*Citrobacter* spp., *C. braakii*<br>*Gibbsiella quercinecans*<br>*Aeromonas hydrophila* subsp. *hydrophila*
77	9,1	0,024	<1	nd	*Pantoea agglomerans*
78	3,9	0,0223	<1	nd	*Citrobacter* spp.<br>*Hafnia alvei*<br>*Pantoea* spp.
79	3,3	0,0214	<1	nd	*Serratia plymuthica, S. entomophila, S. liquefaciens*

80	1,7	3,8	<1	nd	*Serratia liquefaciens*<br>*Hafnia alvei*<br>*Enterobacter amnigenus biovar 1*
81	3,5	0,0123	<1	nd	*Serratia* spp.<br>*Pantoea agglomerans*
82	2,4	1,3	<1	nd	*Rahnella aquatilis*<br>*Pantoea agglomerans*<br>*Enterobacter* spp.

*nd - not detected

Supplementary materials

Table S2. Results of quantitative and qualitative analysis of bacteriological contamination of sewage sludge and digestate samples

**No. of sample**	**Bacterial concentration**			**Presence of *Salmonella***	**Identified species of gram-negative bacteria using biochemical methods**
	**Total mesophilic bacteria [CFU/g]**	***Enterobacteriaceae* [CFU/g]**	***Escherichia coli* [CFU/g]**		
**Sewage sludge samples**					
1	$5{,}7 \times 10^8$	$2{,}2 \times 10^6$	$2{,}3 \times 10^4$	nd*	*Enterobacter cloacae*<br>*Escherichia coli*<br>*Hafnia alvei*<br>*Comamonas jiangduensis*<br>*Raoultella terrigena*
2	$3{,}4 \times 10^8$	$1{,}9 \times 10^6$	$4{,}2 \times 10^4$	nd	*Aeromonas spp.*<br>*Aeromonas salmonicida*<br>*Yersinia frederiksenii*<br>*Klebsiella oxytoca*<br>*Escherichia coli*
3	6×10^6	$1{,}5 \times 10^5$	$1{,}2 \times 10^3$	nd	*Escherichia coli*<br>*Klebsiella oxytoca*<br>*Aeromonas* spp.

4	1,3 x 10^{8}	1,3 x 10^{6}	<1	nd	*Morganella morganii subsp. morganii*
5	3 x 10^{7}	1,5 x 10^{3}	3,8 x 10^{2}	nd	*Escherichia coli*
6	<1	<1	<1	nd	not detected
7	4,3 x 10^{7}	9,7 x 10^{4}	<1	nd	*Alcaligenes faecalis*
8	1,8 x 10^{6}	<1	<1	nd	not detected
9	1,6 x 10^{3}	<1	<1	nd	not detected
**Digestate samples**					
1	6,8 x 10^{8}	2,8 x 10^{6}	9,1 x 10^{4}	detected	*Proteus mirabilis*<br>*Salmonella enterica* subsp. *enterica* serovar Johannesburg<br>*Citrobacter freundii*<br>*Yersinia intermedia*<br>*Escherichia coli*<br>*Yersinia enterocolitica*<br>*Yersinia spp.*<br>*Aeromonas spp.*
2	9,8 x 10^{8}	1,2 x 10^{6}	9,9 x 10^{6}	nd	*Escherichia coli*

3	0,1 x 10^8	1,7 x 10^6	1,1 x 10^2	nd	*Brevundimonas diminuta*<br>*Oligella urethralis*
4	0,9 x 10^8	3,1 x 10^5	<1	nd	not detected
5	0,6 x 10^8	2,1 x 10^7	4,0 x 10^2	nd	*Ignatzschineria indica*<br>*Klebsiella pneumoniae*<br>*Citrobacter* spp.
6	2,3 x 10^8	2,2 x 10^6	6,1 x 10^4	nd	*Citrobacter gillenii*<br>*Citrobacter freundii*<br>*Aeromonas salmonicida*<br>*Escherichia coli*
7	1,2 x 10^8	3,1 x 10^6	0,8 x 10^2	nd	*Citrobacter freundii*
8	0,2 x 10^8	3,0 x 10^6	<1	nd	*Roseomonas cervicalis*
9	1,8 x 10^8	1,5 x 10^7	<1	nd	*Citrobacter braakii*

*nd - not detected
